# Editorial: Aurora Kinases: Classical Mitotic Roles, Non-Canonical Functions and Translational Views

**DOI:** 10.3389/fonc.2017.00048

**Published:** 2017-03-22

**Authors:** Ignacio Pérez de Castro, Mar Carmena, Claude Prigent, David M. Glover

**Affiliations:** ^1^Instituto de Salud Carlos III, Madrid, Spain; ^2^Wellcome Trust Centre for Cell Biology, University of Edinburgh, Edinburgh, UK; ^3^Centre national de la recherche scientifique (CNRS), Rennes, France; ^4^University of Cambridge, Cambridge, UK

**Keywords:** aurora kinase, mitosis, antitumoral therapy, aurora inhibitors, non-canonical roles

**Editorial on the Research Topic**

**Aurora Kinases: Classical Mitotic Roles, Non-Canonical Functions and Translational Views**

Aurora kinases are key mitotic regulators that have also been associated with tumor development and progression. The interest on this highly conserved family of protein kinases has grown exponentially since they were discovered in the 1990s. Despite the steady increase in the number of laboratories involved and the consequent boost of the volume of research output during the last years, the study of Aurora kinases remains a very dynamic area in which new discoveries frequently keep coming to light. From a clinical perspective, the interest on Aurora kinase biology stems from their identification as targets for drug development; an increasing number of Aurora kinase inhibitors are being tested in preclinical projects and clinical trials. In this Frontiers Research Topic, we have aimed to not only review and revisit different aspects of the functions and regulation of Aurora kinases but also provide a forum for the publication of new developments in the field. We have been privileged to count on contributions from authors and reviewers that include some of the most experienced voices in our research area.

In their introductory article to the Research Topic, two old-timers in the field, David Glover and Bill Earnshaw, have provided a historical perspective of Aurora Kinase research. By looking at the field’s origin using genetic screens in *Drosophila* and yeast and cell biological studies in vertebrates that led to the identification of Aurora kinases and their partner proteins, the authors give us a unique first witness account of the development of the field (Carmena et al.).

Several articles in this Research Topic focus exclusively upon a single member of the Aurora kinase family. Among the contributions focused on Aurora A, Garrido and Vernos review the regulation of this kinase by TPX2, discussing its relevance as well as its (weak) conservation throughout evolution and potential key role in tumorigenesis. Two manuscripts from the Guarguaglini and Medema labs explore the interplay between Aurora A kinase and PLK1 and how this contributes to the regulation of different processes in mitosis (Asteriti et al.; Bruinsma et al.). Moving forward to the final stage of mitosis, Reboutier et al. report on the emerging role of Aurora A on the regulation of mitotic exit. The Research Topic also explores the mitotic and non-mitotic roles of Aurora A in the context of oncogenic transformation and tumor progression (D’Assoro et al.); this includes a review on the interactions between the kinase and the tumor suppressor p53 and the possible consequences for their signaling pathways in tumor cells (Sasai et al.). Finally, we have a report on recently described non-canonical roles of Aurora A kinase in DNA replication (Tsunematsu et al.).

On the other side of the coin, among the articles focusing upon Aurora B kinase, two explore its roles in specific stages of mitosis and cytokinesis: Krenn and Musacchio offer a detailed review on the role of Aurora B in chromosome bi-orientation and spindle checkpoint signaling; whereas D’Avino and Capalbo take us through a thorough analysis of the later roles of Aurora kinase within the CPC, focusing on its roles in controlling the assembly of the cleavage furrow, central spindle, and midbody and analyzing the function of the complex in the control of abscission timing. Finally, the chapter by Lindon et al. reviews the ubiquitin-dependent proteolytic regulation of Aurora B in mitosis.

The lesser known member of the Aurora family, Aurora C kinase, is the subject of two reports that center in its role in meiosis (Yang et al.; Quartuccio and Schindler), the latter one also exploring in this context the significance of its expression in cancer cells (Quartuccio and Schindler).

As an important part of this Research Topic, we wanted to include in an overview of different aspects of the use of Aurora kinase as targets for drug development. D’Assoro et al. have reviewed the potential of Aurora A as a therapeutic target in cancer, while Bavetsias and Linardopoulos have summarized the properties of the known Aurora kinase inhibitors currently in the clinic and have discussed current and future directions of such research. The contribution of de Groot and colleagues has given us an invaluable study of 10 commercially available Aurora inhibitors, including a set of “guidelines” for their efficient use in cell biology experiments (de Groot et al.). A group from Lilly Research Laboratories (Marugán et al.) contributes a useful technical manuscript describing phenotypic screening assays to develop Aurora kinase inhibitors. Niu et al. from Takeda Pharmaceuticals review the use of Alisertib (the highly specific Aurora A kinase inhibitor in advanced trials) in cancer therapy. Finally, in this section, the contribution from Nikonova et al. explores the potential of combined therapy directed against both Aurora A and EGFR for the treatment of autosomal-dominant polycystic kidney disease.

We also wanted to have a flavor of some of the non-canonical roles of Aurora kinases outside of mitosis. We have managed to capture some of this activity in the article by Hascoet et al., who have reviewed some of these unconventional functions of Aurora kinases in kidney tumorigenesis.

We hope that the efforts of our authors and referees will find value in the field as we feel that this series of articles “Aurora kinases: classical mitotic roles, non-canonical functions, and translational views” represents an impressive snapshot of our current knowledge of the different functions of Aurora kinases. Written by experts in the field, we hope that this collection will inspire new research projects that will lead to a better understanding of the role of these kinases in cancers.

## Author Contributions

The authors are the editors of the Research Topic “Aurora kinases: classical mitotic roles, non-canonical functions, and translational views.” The four have equally contributed to this editorial.

## Conflict of Interest Statement

The authors declare that the work was conducted in the absence of any commercial or financial relationships that could be construed as a potential conflict of interest.

